# The mediating role of unhealthy behaviors and body mass index in the relationship between high job strain and self-rated poor health among lower educated workers

**DOI:** 10.1007/s00420-020-01565-y

**Published:** 2020-09-05

**Authors:** S. H. van Oostrom, A. Nachat, B. Loef, K. I. Proper

**Affiliations:** Center for Nutrition, Prevention and Health Services, National Institute for Public Health and the Environment, P. O. Box 1, 3720 BA Bilthoven, The Netherlands

**Keywords:** Job strain, Lifestyle, Body mass index, Self-rated health, Worker, Education

## Abstract

**Objectives:**

The objective of this study is to examine the mediating role of unhealthy behaviors and body mass index (BMI) in the relationship between high job strain and self-rated poor health in workers with a low education.

**Methods:**

A total of 8369 low educated workers, who participated in the Lifelines cohort study during the period 2012–2017, were included. Self-reported job strain, health behaviors (smoking, physical activity, and fruit and vegetable consumption), and BMI were assessed at baseline, and self-rated health after 2 years. To assess mediation by the health behaviors and BMI, structural equation modeling with logistic and multinomial regression analyses were performed.

**Results:**

Workers with high job strain had a higher odds of poor health (OR 1.34; 95% CI 1.13–1.60) compared to those with low job strain. Workers with high job strain were more likely to have a lack of physical activity (OR 1.14; 95% CI 1.01–1.28), but were not more likely to smoke, to be overweight or obese, or to have a low fruit or vegetable consumption. Workers who smoke, have a lack of physical activity or are overweight or obese are more likely to report poor health (OR 1.37; 95% CI 1.16–1.60, OR 1.25; 95% CI 1.08–1.43, OR 1.37; 95% CI 1.16–1.61, OR 2.25; 95% CI 1.86–2.72). Indirect (mediating) effects of unhealthy behaviors and BMI in the relationship between high job strain and poor health were small and not statistically significant.

**Conclusions:**

No mediating effects of unhealthy behaviors or BMI were found in the relationship between high job strain and self-rated poor health among workers with a low educational level.

## Introduction

Socio-economic health inequalities are substantial, emphasized by poor health outcomes and more mortality for people with a low socio-economic position (SEP) compared to those with a high SEP (Gallo et al. 2012; Marmot 2005). Over the years, socio-economic inequalities in (self-rated) health persist and in some countries have even widened over time (Mackenbach 2012). Tackling of the health inequalities is a priority in many European countries. It is assumed that no single factor can explain health inequalities (Bambra et al. 2010) and there is support that material factors, physical and psychosocial working conditions, and health behaviors all contribute to these inequalities (Moor et al. 2017). In their recent review, Dieker et al. systematically summarized the factors explaining socio-economic inequalities in self-rated health among workers and concluded that both psychosocial and physical working conditions and lifestyle factors have a substantial role (Dieker et al. 2019). The authors concluded the contribution of work factors to inequalities in self-rated health to explain over one-third of the socio-economic inequalities in self-rated health among workers and a somewhat smaller contribution (i.e., one-fifth) of lifestyle factors (Dieker et al. 2019). Still, the question on the interrelationship between working conditions, unhealthy behavior, and poor self-rated health was not investigated in this review.

Poor psychosocial working conditions, such as high job strain, might shape unhealthy behavior by adversely affecting workers’ food choices, levels of physical activity, or alcohol consumption patterns (LaMontagne 2012; Stansfeld and Marmot 2002). It has been hypothesized that stressful jobs characterized by high psychological demands and low control (high job strain) result in fatigue and greater need for recovery, increasing the likelihood of physical inactivity and another hypothesis proposed that passive, unchallenging jobs with high demands and little control over work can lead to reduced self-efficacy, which in turn may result in more passive lifestyles (Fransson et al. 2012a). These hypotheses were confirmed in previous studies and reviews, showing that workers with high job strain were more likely to be physically inactive (Fransson et al. 2012a; Kouvonen et al. 2005a; Lallukka et al. 2008) and smoked more cigarettes (Heikkila et al. 2012b; Kouvonen et al. 2005b). In a large individual meta-analysis including more than 45,000 workers who were physically active at baseline, the odds of becoming physically inactive were 21% higher for those with high job strain (Fransson et al. 2012a). Among (former) smokers high job strain was associated with a higher likelihood of being a current smoker at follow-up compared to those with low job strain (Kouvonen et al. 2005b). Heikkilä et al. showed that workers with job strain were about 25% more likely than those with no job strain to have an unhealthy lifestyle for at least one out of four of the following lifestyle factors: body mass index (BMI), smoking, alcohol consumption, and physical activity (Heikkilä et al. 2013).

As workers with a low educational level often have poor psychosocial working conditions, and are more likely to engage in unhealthy behavior and to have a poor health status, several authors refer to the need for longitudinal studies to provide more insight in the mechanism underlying the relationship between specific working conditions, such as job strain, unhealthy behavior, and self-rated poor health among workers with a low educational level (Dieker et al. 2019; Lallukka et al. 2008; LaMontagne 2012; Mustard et al. 2003). We hypothesize that workers with high job strain report a poorer health status which is partly explained by unhealthy behavior and unhealthy weight. As this has not been examined so far, the objective of this study was to examine the mediating role of unhealthy behaviors and BMI in the relationship between high job strain and self-rated poor general health among workers with a low educational level.

## Materials and methods

### Study population and study design

**Fig. 1:**
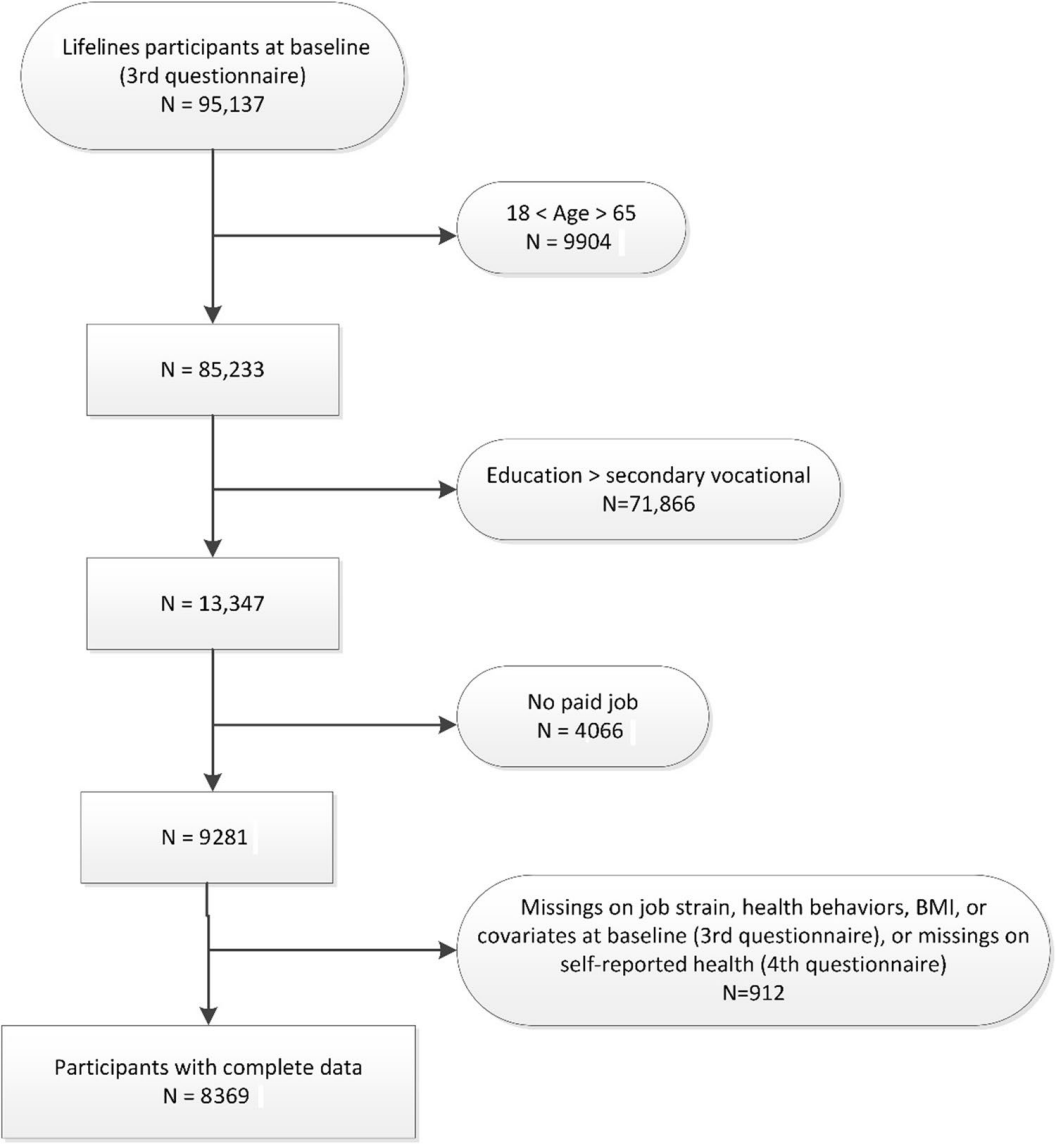
Flow chart of study population

Lifelines is an ongoing multi-disciplinary prospective population-based cohort study examining in a unique three-generation design the health and health-related behaviors of 167,729 persons living in the North of The Netherlands (Scholtens et al. 2014). It employs a broad range of biomedical, socio-demographic, behavioral, physical, and psychological factors which contribute to the health and disease of the general population, with a special focus on multi-morbidity and complex genetics. Participants were recruited between 2006 and 2013, including a questionnaire and a physical examination. Follow-up questionnaires were sent every 1.5 years. Details of the Lifelines study design are reported elsewhere (Scholtens et al. 2014). As psychosocial working conditions, including job strain, were measured in the third follow-up questionnaire (between 2012 and 2015, *n* = 95,137), this was considered as the baseline measurement for this study. Participants over 65 years of age, and younger than 18 years (*n* = 9904), as well as those with the highest completed level of education (at either baseline or follow-up) of secondary vocational education or work-based learning pathway or higher (*n* = 71,886), and those without a paid job at baseline (*n* = 4066) were excluded from this study, resulting in 9281 workers with a low educational level. Of those workers, 90.2% had complete data on job strain, three potential mediators (smoking, lack of physical activity, overweight, and obesity) and covariates at baseline, and self-reported health at follow-up (i.e., the fourth questionnaire, 2014–2017), which resulted in a study population of in total 8369 workers (Fig. 1). The Lifelines study was approved by the medical ethical committee of the University Medical Center Groningen. Written informed consent was acquired from all participants.

### Variables

#### Job strain

Psychosocial job strain was measured by eight items of the validated second version of the Copenhagen Psychosocial Questionnaire (COPSOQ II) (Pejtersen et al. 2009). High job strain was defined as high job demands and low job control following the job-demand-control model (Theorell 1996). Job demands were measured by two items on work pace and two items on quantitative demands. Job control was measured by two items about influence at work and two items about possibilities for development. Answers were given on a 5-point scale from always to (almost) never. For both scales, the scores were summed and dichotomized on basis of the median (Fransson et al. 2012b). The presence of job strain was defined as having high job demands (i.e., higher than the median of the job demand scores) and a low job control (i.e., lower than the median of the job control scores).

#### Unhealthy behaviors and BMI

Physical activity was assessed using one question from the validated SQUASH (“On average how many days per week do you bicycle, perform odd jobs, gardening or exercise, for all activities added together, for at least half an hour?”) (Wendel-Vos et al. 2003). Responses were dichotomized into meeting versus not meeting the recommended frequency of physical activities in public health recommendations (≥ 5 days/week vs < 5 days/week) (Kemper et al. 2000). Smoking was measured using a single question (“Do you smoke now, or have you smoked in the past month?”) to be answered on a dichotomous scale (yes/no). BMI (kg/m^2^) was calculated based on self-reported weight and measured length, and categorized into healthy body weight (18.5–24.9 kg/m^2^), overweight (25–29.9 kg/m^2^), and obesity (≥ 30 kg/m^2^) (WHO 2000).

Fruit and vegetable consumption were assessed in a random subsample of the study population, as part of the extensive validated Dutch National Food Consumption Survey. Two single-item questions were included in the questionnaire asking to the frequency of the fruit or vegetable consumption in the past month (Ocké et al. 1997). Response options were on a 7-point scale (“not this month” to “6–7 days a week”). Fruit and vegetable consumption were both dichotomized into meeting versus not meeting the recommendations (6–7 days/week vs < 6–7 days/week) (Gezondheidsraad 2015).

#### Self-rated health

Perceived general health was assessed at follow-up based on one item of the validated RAND 36-item Health Survey (van der Zee and Sanderman 2012). The question was phrased as “In general, would you say your health is …?” with response categories on a 5-point scale. Responses were dichotomized into good health (excellent, very good, and good) and poor health (fair, poor). This self-rated health measure is widely used and has been shown a good predictor of mortality and morbidity (Jylha 2009; Robine et al. 2003).

#### Covariates

Covariates consisted of age, sex, and the number of working hours per week. The number of working hours was assessed by the question “how many hours do you do paid work on average per week?”

### Statistical analyses

**Fig. 2:**
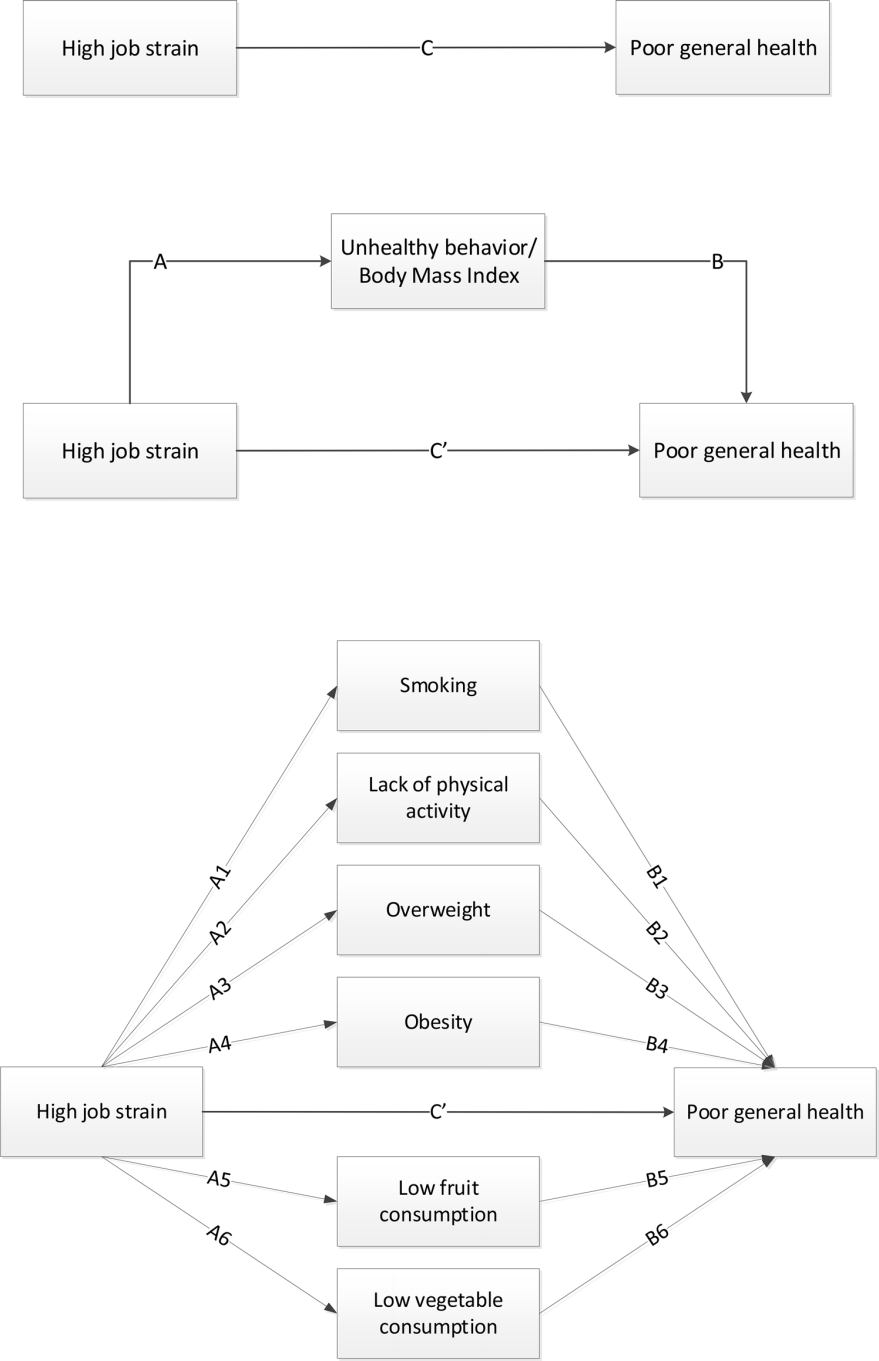
Univariable (middle diagram) and multiple mediation (lower diagram) model of the total effect of high job strain on poor general health (*c*, upper diagram), the effects of high job strain on unhealthy behavior (*a*-paths), the effects of unhealthy behavior on poor health (*b*-paths), and the direct effect of high job strain (*c*’) on poor health

Descriptive statistics were used to present baseline characteristics of the study population stratified for workers with a good and poor health. To examine the mediating role of unhealthy behavior or BMI in the association between job strain and general health, structural equation modeling (SEM) was used, in which job strain, unhealthy behaviors, BMI, and socio-demographic characteristics were assessed at baseline and self-rated general health was assessed at follow-up (Fig. 2). In the models, the total effect (*c*-path) of job strain on general health, the effects of job strain on health behaviors and BMI (*a*-paths), the effects of health behaviors and BMI on general health (*b*-paths), and the direct effects of job strain on self-rated health, independent from the mediator (*c*’-path), were determined by means of logistic and multinomial (for BMI) regression analyses. All analyses were adjusted for age, sex, and working hours per week. First, univariable mediation models were performed for potential mediators. Then, a multiple mediation model, i.e., including all potential mediators, was performed. Indirect effects indicate the extent to which the effect of high job strain on self-rated poor health was mediated by each unhealthy behavior or BMI, and were calculated by the product of the *a*- and *b*-paths. Bootstrapping using 5000 bootstrap resamples was used to calculate 95% confidence intervals for the indirect effects (Preacher and Hayes 2008; Sobel 1982).

All 8369 workers with complete data on job strain, three potential mediators (smoking, lack of physical activity, overweight and obesity), self-rated health, and covariates were included in a first mediation analysis. Since a random subsample of the study population received questions on fruit and vegetable consumption, a second mediation analysis was performed for 5568 workers with complete data on the variables of interest and all potential mediators, i.e. fruit and vegetable consumption. Statistical analyses were performed using IBM SPSS Statistics 23 and StataMP 13.

## Results

### Study population

The mean age of the 8369 workers with a low educational level was 49.9 (SD 7.8) years, 46.2% was male, and participants worked on average 30 (SD 13.7) hours per week (Table 1). 16.6% of the workers reported high job strain, 21.7% smoked, almost half of the workers (47.3%) did not adhere to physical activity recommendations, 44.8% was overweight and 16.4% obese, almost two-third (64.9%) reported a low fruit consumption, and 83.7% reported a low vegetable consumption. Among workers in self-rated poor health, 20.2% reported high job strain, 26.0% smoked, and 25.4% was obese, compared to 16.2% with high job strain, 21.2% who smoked and 15.2% with obesity among those in good health (Table 1).

**Table 1:** Socio-demographic and lifestyle baseline characteristics of workers with a low educational level (*N* = 8369)

		Total *N* = 8369^a^	Good self-rated health *N* = 7445 (89.0%)	Poor self-rated health *N* = 924 (11.0%)
Sex	*N*,% men	3868 (46.2)	3452 (46.4)	416 (45.0)
Age (in years)	Mean, SD	49.9 (7.8)	49.9 (7.8)	49.7 (7.6)
Working hours	Mean, SD	30.0 (13.7)	30.2 (13.7)	28.4 (13.9)
High job strain	*N*, %	1392 (16.6)	1203 (16.2)	189 (20.5)
Health behaviors				
Smoking	*N*, %	1820 (21.7)	1580 (21.2)	240 (26.0)
Lack of physical activity^b^	*N*, %	3960 (47.3)	3474 (46.7)	486 (52.6)
Overweight^c^	*N*, %	3747 (44.8)	3334 (44.8)	413 (44.7)
Obesity^c^	*N*, %	1370 (16.4)	1135 (15.2)	235 (25.4)
Low fruit consumption^d^	*N*, %	3612 (64.9)	3183 (64.4)	429 (69.0)
Low vegetable consumption^d^	*N*, %	4661 (83.7)	4138 (83.7)	523 (84.1)

*SD* standard deviation

^a^A random subsample of the study population (*N* = 5568) had data on fruit and vegetable consumption, the frequencies and means for the socio-demographic and lifestyle baseline characteristics did not differ substantially between the total study population (*N* = 8369) and the subsample (*N* = 5568) (all differences in frequencies were < 0.7%)

^b^Lack of physical activity is defined by being physically active on < 5 days a week

^c^Overweight is defined as a BMI of 25–29.9 kg/m^2^ and obesity as a BMI of ≥ 30 kg/m^2^

^d^Low fruit consumption or vegetable consumption is defined as fruit/vegetable consumption at < 6 days a week

### Mediation model 1 (three unhealthy behaviors, excluding fruit and vegetable consumption)

On the basis of 8369 workers with a low educational level, the total effect (*c*-path) of high job strain on self-rated poor health was an odds ratio (OR) of 1.34 (95% confidence interval (CI) 1.13–1.60), adjusted for age, sex, and working hours (Table 2). This indicated that workers with a high job strain are more likely to report poor general health compared to those with a low job strain. The multiple mediation model for the relationship between high job strain and unhealthy behavior (*a*-path) showed that workers with high job strain more often reported a lack of physical activity (OR 1.14; 95% CI 1.01–1.28), compared to workers with low job strain. No statistically significant relations between job strain and smoking or BMI were found. As to the *b*-paths of the multiple mediation model, significant effects were shown for smoking (OR 1.37; 95% CI 1.16–1.60), lack of physical activity (OR 1.25; 95% CI 1.08–1.43), overweight (OR 1.37; 95% CI 1.16–1.61), and obesity (OR 2.25; 95% CI 1.86–2.72) with self-rated poor health at follow-up (Table 2). The direct effect of job strain on general health (*c*’-path) was an OR of 1.32 (95% CI 1.11–1.57). The ORs for the indirect effects (*a* × *b*) ranged between 0.98 (for smoking) and 1.14 (for obesity) and were all non-significant, indicating that the effect of high job strain on self-rated poor health was not mediated by unhealthy behavior or BMI.

**Table 2:** Path coefficients of the univariable and multiple mediation model (expressed as odds ratios) of unhealthy behavior and BMI in the relation between high job strain and poor self-rated health among 8369 low educated workers

	*c*-path (total effect)	*c*’-path (direct effect)	*a*-path (high job strain > unhealthy behavior)	*b*-path (unhealthy behavior > poor health)	*a *× *b *(indirect effects)^a^
	OR (95% CI)	OR (95% CI)	OR (95% CI)	OR (95% CI)	OR (95% CI)
Total effect	**1.34 (1.13–1.60)**				
Univariable mediation model					
Smoking		**1.35 (1.14–1.60)**	0.95 (0.82–1.09)	**1.32 (1.13–1.55)**	0.99 (0.94–1.02)
Lack of physical activity		**1.33 (1.12–1.59)**	**1.14 (1.01–1.28)**	**1.32 (1.15–1.52)**	**1.04 (1.00–1.08)**
BMI		**1.33 (1.12–1.58)**			
Overweight			0.97 (0.85–1.10)	**1.37 (1.16–1.61)**	0.99 (0.95–1.03)
Obesity			1.17 (0.99–1.38)	**2.24 (1.86–2.71)**	1.14 (0.99–1.31)
Multiple mediation model		**1.32 (1.11–1.57)**			
Smoking			0.94 (0.82–1.09)	**1.37 (1.16–1.60)**	0.98 (0.93–1.03)
Lack of physical activity			**1.14 (1.01–1.28)**	**1.25 (1.08–1.43)**	1.03 (0.99–1.06)
BMI					
Overweight			0.97 (0.85–1.10)	**1.37 (1.16–1.61)**	0.99 (0.95–1.03)
Obesity			1.17 (0.99–1.38)	**2.25 (1.86–2.72)**	1.14 (0.99–1.30)

Analyses were adjusted for working hours, age, and sex

ORs given in bold indicate statistical significance

*OR *odds ratio, *CI* confidence interval

^a^Indirect effects are calculated by taking the product of the *a*-paths and the *b*-paths (natural logarithms) (e.g., (*a*^2^ × *b*^2^) =* e*^(0.111 × − 0.284)^ = 0.97)

### Mediation model 2 (five unhealthy behaviors, including fruit and vegetable consumption)

**Fig. 3:**
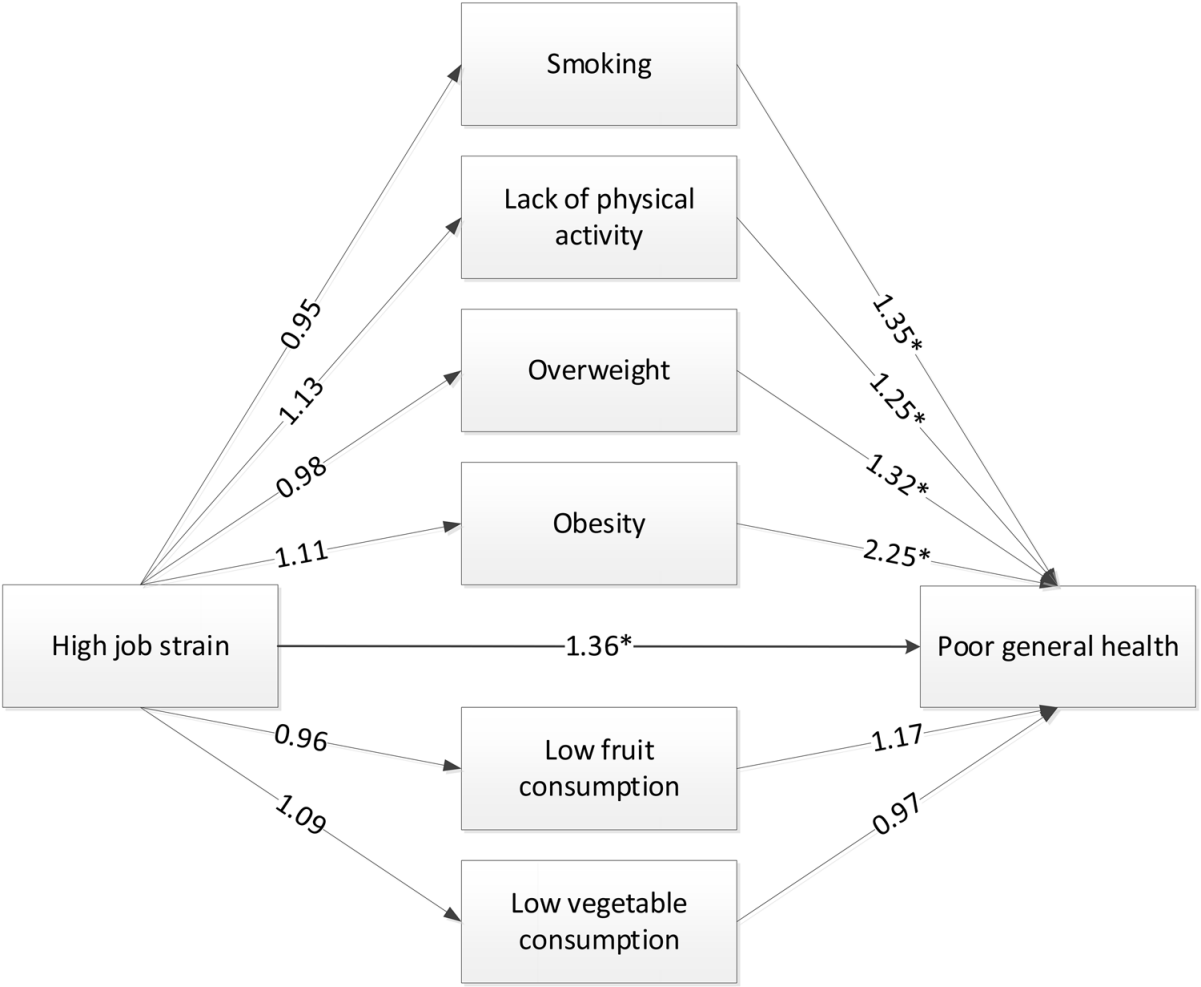
The multiple mediation model including the odds ratio’s (ORs) for the *a*-paths of high job strain on the unhealthy behaviors and BMI, the *b*-paths of the unhealthy behaviors on poor general health, and the direct effect of high job strain on poor general health (*N* = 5568 workers with a low educational level). All coefficients are adjusted for age, sex, and working hours. Statistical significance of the OR is indicated by asterisk

Among the 5568 workers with a low educational level, the total effect (*c*-path) of high job strain on self-rated poor health was an OR of 1.38 (95% CI 1.12–1.70), adjusted for age, sex, and working hours (Table 3). The multiple mediation model showed no statistically significant relations between job strain and unhealthy behaviors (*a*-paths). Again, the *b*-paths of the multiple mediation model showed significant effects for smoking (OR 1.35; 95% CI 1.11–1.64), lack of physical activity (OR 1.25; 95% CI 1.05–1.49), overweight (OR 1.32; 95% CI 1.08–1.61), and obesity (OR 2.25; 95% CI 1.78–2.84) with self-rated poor health at follow-up (Table 3; Fig. 3), but not for low fruit and vegetable consumption. The direct effect of job strain on general health (*c*’-path) was an OR of 1.36 (95% CI 1.10–1.68). The ORs for the indirect effects (*a* × *b*) were close to 1.00 and ranged between 0.99 (for smoking, overweight, and low fruit and vegetable consumption) and 1.09 (for obesity) and were all non-significant.

**Table 3:** Path coefficients of the univariable and multiple mediation model (expressed as odds ratios) of unhealthy behavior and BMI in the relation between high job strain and poor self-rated health among 5568 low educated workers

	*c*-path (total effect)	*c*’-path (direct effect)	*a*-path (high job strain > unhealthy behavior)	*b*-path (unhealthy behavior > poor health)	*a *× *b *(indirect effects)^a^
	OR (95% CI)	OR (95% CI)	OR (95% CI)	OR (95% CI)	OR (95% CI)
Total effect	**1.38 (1.12–1.70)**				
Univariable mediation model					
Smoking		**1.38 (1.12–1.70)**	0.95 (0.80–1.13)	**1.35 (1.11–1.63)**	0.99 (0.93–1.04)
Lack of physical activity		**1.37 (1.11–1.68)**	1.13 (0.98–1.30)	**1.36 (1.11–1.68)**	1.03 (0.99–1.10)
BMI		**1.37 (1.11–1.68)**			
Overweight			0.98 (0.83–1.14)	**1.33 (1.09–1.62)**	0.99 (0.94–1.04)
Obesity			1.12 (0.91–1.37)	**2.27 (1.81–2.86)**	1.09 (0.92–1.31)
Low fruit consumption		**1.38 (1.12–1.70)**	0.96 (0.83–1.12)	**1.29 (1.07–1.55)**	0.99 (0.95–1.03)
Low vegetable consumption		**1.38 (1.12–1.69)**	1.09 (0.90–1.33)	1.06 (0.84–1.33)	1.00 (0.97–1.05)
Multiple mediation model		**1.36 (1.10–1.68)**			
Smoking			0.95 (0.80–1.13)	**1.35 (1.11–1.64)**	0.99 (0.93–1.04)
Physical inactivity			1.13 (0.98–1.30)	**1.25 (1.05–1.49)**	1.03 (0.99–1.08)
BMI					
Overweight			0.98 (0.83–1.14)	**1.32 (1.08–1.61)**	0.99 (0.94–1.04)
Obesity			1.11 (0.91–1.37)	**2.25 (1.78–2.84)**	1.09 (0.92–1.31)
Low fruit consumption			0.96 (0.83–1.12)	1.17 (0.96–1.41)	0.99 (0.96–1.02)
Low vegetable consumption			1.09 (0.90–1.33)	0.97 (0.77–1.23)	0.99 (0.96–1.03)

Analyses were adjusted for working hours, age, and sex

ORs given in bold indicate statistical significance

*OR* odds ratio, *CI* confidence interval

^a^Indirect effects are calculated by taking the product of the *a*-paths and the *b*-paths (natural logarithms) (e.g., (*a*^2^ × *b*^2^) =* e*^(0.111 × − 0.284)^ = 0.97)

## Discussion

In the present study, it was hypothesized that unhealthy behaviors and BMI may partly mediate the relationship between high job strain and self-rated poor health among workers with a low education. The findings confirmed that workers with high job strain are more likely to perceive a poor health, but except for physical activity in model 1, not more likely to engage in unhealthy behavior or to have overweight. Workers who smoked, had a lack of physical activity, and were overweight or obese were more likely to perceive poor health, but this is not the case for those with a low fruit or vegetable consumption. Health behaviors and BMI were not mediators in the relationship between high job strain and poor health, indicated by the small and non-significant indirect effects.

To the best of our knowledge, no previous study has examined the potential mediating effect of unhealthy behaviors in the association between high job strain and self-rated poor health in workers with a low educational level. The relationship between job strain and health behaviors is well documented among workers in general, as the IPD-Work (individual-participant data meta-analysis of working populations) Consortium published five meta-analyses on this relationship (Fransson et al. 2012a; Heikkila et al. 2012a, b, 2013; Nyberg et al. 2012). The overall conclusions of these meta-analyses based on cross-sectional analyses in European studies confirm an association between high job strain and unhealthy behaviors. Current smokers had a higher odds (OR 1.11; 95% CI 1.03–1.18) of high job strain than never smokers (Heikkila et al. 2012b) and workers with obesity had a higher odds (OR 1.07; 95% CI 1.02–1.12) for high job strain compared to those with a healthy weight (Nyberg et al. 2012). Although the pooled ORs in the meta-analyses were reported for the association between unhealthy behavior and job strain and this was opposite to the direction of the association in the mediation model in our study, the ORs were obtained from cross-sectional analyses and are therefore relevant for the hypothesized relationship between high job strain, unhealthy behavior, and poor health. Further, workers with high job strain have a higher odds (OR 1.26; 95% CI 1.15–1.38) for physical inactivity in leisure time compared to workers with low job strain (Fransson et al. 2012a). Among workers with a low SEP and high job strain, the odds ratio for physical inactivity in leisure time was 1.31 (95% CI 1.22–1.41) (Fransson et al. 2012a). The relationship between high job strain and physical inactivity was confirmed by the findings in the present study, but the relationships between high job strain and smoking or obesity were not confirmed. As the present study focused on workers with a low educational level and the meta-analyses for smoking and BMI were not stratified but adjusted for educational level, the findings are difficult to compare. Further, Nyberg et al. concluded a U-shaped cross-sectional association between job strain and BMI, with significant higher ORs for both the categories of underweight and obesity (Nyberg et al. 2012). In the present study, the relationship between job strain and obesity was in the same direction, but still because of differences in the study population (size), not fully comparable with the meta-analysis. Furthermore, differences in the assessment of health behaviors, the specific questions that were taken into account to define job demands and control, and the binary or categorical job strain variables may explain different results for the relationship between job strain and health behaviors. We found no significant relationship between high job strain and low fruit and vegetable consumption, except for the univariable model for low fruit consumption. This specific relationship was not studied by the IPD Work Consortium. Other studies investigating the association between job strain and healthy diet, including fruit and vegetable consumption, are sparse and results are inconsistent (Goston et al. 2013; Lallukka et al. 2004; Payne et al. 2005).

Most effects of unhealthy behaviors and BMI on general self-rated poor health (*b*-path) were statistically significant. Smoking, overweight, obesity, and physical inactivity were related with a poorer health and thereby confirmed the evident health impact of these health behaviors (Borg and Kristensen 2000; Hämmig and Bauer 2013; Molarius et al. 2007). Low fruit and vegetable consumption were not associated with poor health at follow-up in the multivariable model. A recent Canadian study also concluded no association between inadequate fruit and vegetable consumption and self-rated health, whereas the association with self-rated health was confirmed for other health behaviors (Riediger et al. 2019).

Small, non-significant indirect effects and the observation that the direct effect of job strain on general health (*c*’-path) was very similar to the total effect (*c*-path) indicate that the pathway via unhealthy behaviors and BMI was not supported by our data and may not be the main pathway the relation between high job strain and self-rated poor health follows. These findings were contrary to our hypothesis and those of others who stated that mediation by health behaviors and BMI in the relationship between high job strain and self-rated poor health is plausible (Lallukka et al. 2008; LaMontagne 2012). Instead, Lallukka et al. noticed that associations between job strain and health behaviors were generally weak and therefore argued that this may provide only modest support for potential mediation of the effect of high job strain on workers’ health by unhealthy behaviors (Lallukka et al. 2008). The weak associations between high job strain and unhealthy behaviors appeared to be the main reason a mediating role of health behaviors or BMI was not supported in our study. Yet, there is little knowledge on the mechanism of job strain leading to unhealthy behaviors. A proposed mechanism is that job stress leads to reduced self-efficacy, which in turn may result in unhealthy behavior (Fransson et al. 2012a; LaMontagne 2012). It may also be plausible that the relationship between job strain and health behaviors is different for workers with a high and low education or SEP, as the impact of high job strain might differ between them (LaMontagne 2012). Therefore, we recommend future studies to first increase our understandings about the relationship between job strain and unhealthy behaviors. In the meantime, based on the associations found between the health behaviors and BMI on self-rated health (*b*-paths) and the direct effects of job strain on self-rated health (*c*’ path), promoting favorable psychosocial working conditions and a healthy lifestyle among workers with a low educational level is recommended.

A strength of the current study is the use of the Lifelines cohort study, which includes a representative population (Klijs et al. 2015). The large sample size and prospective design of the Lifelines cohort enabled us to study the mediation of unhealthy behavior in the relationship between high job strain and self-rated poor health prospectively and among workers with a low educational level. With the longitudinal design we could study self-rated health at a later moment in time than the exposure and the mediating factor. The exposure and potential mediator were measured at the same measurement, as it is realistic to expect an immediate change in the lifestyle behavior when exposed to high job strain rather than after 2 years. Despite the prospective mediation analysis reverse causation cannot be ruled out, since it is known that workers with poor self-rated health more often have poor working conditions and unhealthy behaviors, especially among workers with a low educational level (Dieker et al. 2019). As the Lifelines study arranges a broad data collection on several domains, we were able to study different health behaviors as mediating factors, smoking, physical inactivity, and BMI, and for a subsample of the population also low fruit and vegetable consumption. Questions about fruit and vegetable consumption were part of an extensive Food Frequency Questionnaire, which is a long and time-consuming questionnaire. Therefore, a flower FFQ design was developed which means that participants of the Lifelines study received random parts of the FFQ-questionnaire during follow-up (Brouwer-Brolsma et al. 2017); as a result, the items for fruit and vegetable consumption were not available for all participants. Further, in general the measurement of the self-reported health behaviors was very broad. For fruit and vegetable consumption, for instance, the questions did not include the amount but only the frequency of consumed fruit and vegetables in the past month. Also for physical activity, the formulation of the specific question was very broad and referred to the number of days a week a participant bicycled, performed odd jobs, gardened, or exercised for at least half an hour per day. Based on this question we could roughly categorize participants according to the former public health recommendations in The Netherlands (Kemper et al. 2000). However, more information about the duration and intensity of activities is preferable. The lack of detail in the questions for the health behaviors may have resulted in incorrect categorization of participants with regard to the adherence to recommendations for healthy lifestyles, but a different response to the lifestyle questions for workers with a high and low job strain is not expected. About 16% of the workers in our study population had high job strain, this prevalence of high job strain may seem quite low but is very similar compared to other studies on job strain showing a range of 12% to 22% with high job strain (Fransson et al. 2012a).

## Conclusion

The findings of this study do not support a mediating effect of unhealthy behaviors or BMI in the relationship between high job strain and self-reported poor general health among workers with a low educational level. Despite this, the findings confirm that workers with a low educational level and high job strain, as well as workers with a low educational level who smoke, have a lack of physical activity or are overweight or obese, are more likely to report poor health. As this is the first study to the mediating effect of unhealthy behaviors in the relationship between job strain and self-rated health, future research is needed.
